# SAbPred: a structure-based antibody prediction server

**DOI:** 10.1093/nar/gkw361

**Published:** 2016-04-29

**Authors:** James Dunbar, Konrad Krawczyk, Jinwoo Leem, Claire Marks, Jaroslaw Nowak, Cristian Regep, Guy Georges, Sebastian Kelm, Bojana Popovic, Charlotte M. Deane

**Affiliations:** 1 Department of Statistics, Oxford Protein Informatics Group, University of Oxford, 24-29 St Giles', Oxford, OX1 3LB, UK; 2 Pharma Research and Early Development, Large Molecule Research, Roche Innovation Center Munich, Nonnenwald 2, 82377, Penzberg, Germany; 3 Informatics Department, UCB Pharma, 208 Bath Road, Slough, SL1 3WE, UK; 4 Antibody Discovery and Protein Engineering, Medimmune Ltd, Granta Park, Cambridge, CB21 6GH, UK

## Abstract

SAbPred is a server that makes predictions of the properties of antibodies focusing on their structures. Antibody informatics tools can help improve our understanding of immune responses to disease and aid in the design and engineering of therapeutic molecules. SAbPred is a single platform containing multiple applications which can: number and align sequences; automatically generate antibody variable fragment homology models; annotate such models with estimated accuracy alongside sequence and structural properties including potential developability issues; predict paratope residues; and predict epitope patches on protein antigens. The server is available at http://opig.stats.ox.ac.uk/webapps/sabpred.

## INTRODUCTION

Antibodies are proteins that form part of the natural immune system's arsenal. They are now also widely used as a therapeutic modality (1). Methods for generating therapeutic antibody molecules either isolate antigen specific B-cells from an *in vivo* response or screen against a repertoire of molecules using *in vitro* display technologies (2–4). Often, a degree of engineering is required to further optimize molecules for certain therapeutic and manufacturing properties (5). Rational engineering decisions can be informed by knowledge of the structural properties of the molecule. Such properties include which residues on the antibody form contacts with the antigen (paratope) or whether patches are present on the molecule's surface that could cause aggregation. In the absence of an experimentally determined structure, a toolbox of computational methods are required to predict such features (6).

Computational tools that deal with a range of individual antibody informatics problems are available (7). One commonly used tool is for the application of numbering schemes to antibody variable domain sequences (8–10). These annotations allow for sequences to be compared at equivalent positions and make possible the recognition of the complementary determining regions (CDRs) (segments of the antibody that normally contain most of the antigen contact residues). CDR recognition is the first stage of predicting the structure of the variable domains of the antibody, VH and VL, collectively the Fv.

Antibody Fv modelling can be performed with high accuracy (11,12) and provides a fast method for obtaining structural information about a molecule. Models of the antibody Fv can be used in many other ways including paratope prediction (13,14), epitope prediction (15,16) and protein docking (17). These algorithms give information about the specific residues involved in the antibody–antigen interaction and aid decisions about which mutations can be made to enhance or at least not disrupt binding properties. Structural insights gained through modelling also allow potential issues with *in vitro* development to be identified and overcome (5). As the quality of a subsequent prediction is dependent on the quality of the structural information used (14,15), it is important to understand how accurate a model might be especially when it has been generated automatically.

Our SAbPred webserver is a user friendly interface that provides a single platform for structure-based tools useful for the antibody design process. Currently four applications are available: sequence numbering (18); Fv modelling including accuracy estimation and developability annotations; paratope residue prediction (14); and epitope patch prediction (15). An overview of each algorithm is given in the following sections.

## MATERIALS AND METHODS

### Sequence numbering: ANARCI

Numbering schemes annotate equivalent positions in multiple sequences. The ANARCI tool (18) aligns an input sequence to a set of Hidden Markov Models that describe the germline sequences of different types of variable domains from a number of species. The best scoring alignment is translated into one of five commonly used numbering schemes: Kabat (19), Chothia (20), Enhanced Chothia (8), IMGT (21) or AHo (22). ANARCI is able to number both antibody sequences and TCR sequences.

### Fv modelling: ABodyBuilder

SAbPred can automatically model the Fv structure of an antibody using our ABodyBuilder algorithm. The program builds a model from the amino-acid sequence and calculates an estimated accuracy for segments of the model. In brief, a submitted antibody sequence is numbered using ANARCI and the CDR and framework regions are recognized. Templates for the VH and VL framework regions are chosen from SAbDab (23) and orientated with respect to each other using ABangle (24). FREAD (25) is used with CDR specific databases to predict the CDR conformations. If a knowledge-based prediction is not possible then MODELLER (26) is used to model the CDR loop. Finally, SCRWL4 (27) is used to predict the conformations of side chains whose coordinates cannot be copied directly from a template structure.

Models built by ABodyBuilder are of similar quality to other methods included in the most recent Antibody Modelling Assessment (AMA-II) (12) (Supplementary Figure S1). To replicate the blind test conditions of the competition as far as possible, all structures that were released to the PDB after 31 March 2013 were omitted from the template and FREAD databases. The average RMSD for the whole Fv for our models over all 11 targets in AMA-II was 1.19Å; this is comparable to other publicly available pipelines: RosettaAntibody (28) (1.12Å), Kotai Antibody Builder (29) (1.06Å) and PIGS (30) (1.54Å).

### Paratope prediction: Antibody i-Patch

Residues that the antibody uses to make interactions with its specific antigen form the paratope of the molecule. In most cases these residues belong to one of the CDR structural loops but residues outside these regions can also form contacts. SAbPred uses the Antibody i-Patch algorithm (14) to perform paratope prediction. It takes as input the structure of the antigen and the structure or model of the antibody Fv. As output, each residue is annotated with a score. The score describes how often the residue type in its local environment (patch) is involved in antigen binding in known structures.

Antibody i-Patch has been developed to identify a small set of residues which are highly likely to be part of a paratope and are energetically important for the antibody-antigen recognition. When tested on a non-redundant dataset of antibody–antigen structures Antibody i-Patch achieved 77% precision at a recall of 10% (14). In terms of the ranking returned by Antibody i-Patch, a user should expect three of top five residues and five of the top ten residues to form part of the paratope. These residues are also likely to be among the most energetically important (14). Lower precision but higher recall paratope predictions can be achieved using CDR definitions (typically around 30 and 90%, respectively) or using other prediction methods (13).

### Epitope prediction: EpiPred

Residues that the antibody interacts with on an antigen form the epitope. SAbPred uses the EpiPred algorithm (15) to predict the epitope on a protein antigen for a specific antibody. The algorithm takes as input the structure of the antigen and the structure or model of the antibody. A ranked list of sets of residues, patches, that may form the epitope are returned.

Predictions are made by analyzing the propensity of residues in their given environment to form epitopes in the known structural data. The higher the number of preferable interactions a patch on the antigen can make simultaneously with the antibody paratope, the better its ranking as a potential epitope. When tested on a non-redundant dataset of 30 antibody–antigen structures EpiPred achieved 44% recall at 14% precision (15). A comparison to one of the leading conformational B-cell predictors, DiscoTope 2.0 (16), showed EpiPred's predictions were better on 17 targets, worse on eight and neither of the methods produced a usable prediction on the remaining five.

## INTERFACE AND USAGE

The SAbPred interface can be accessed at http://opig.stats.ox.ac.uk/webapps/sabpred. The front page of the website allows a user to view all completed and running jobs. From here, or using the menu at the top of each page, one may navigate to the different antibody structure based applications described below. No login is required and users are encouraged to take note of the results link provided after submission of their job for later retrieval.

### Sequence numbering

**Figure 1.**
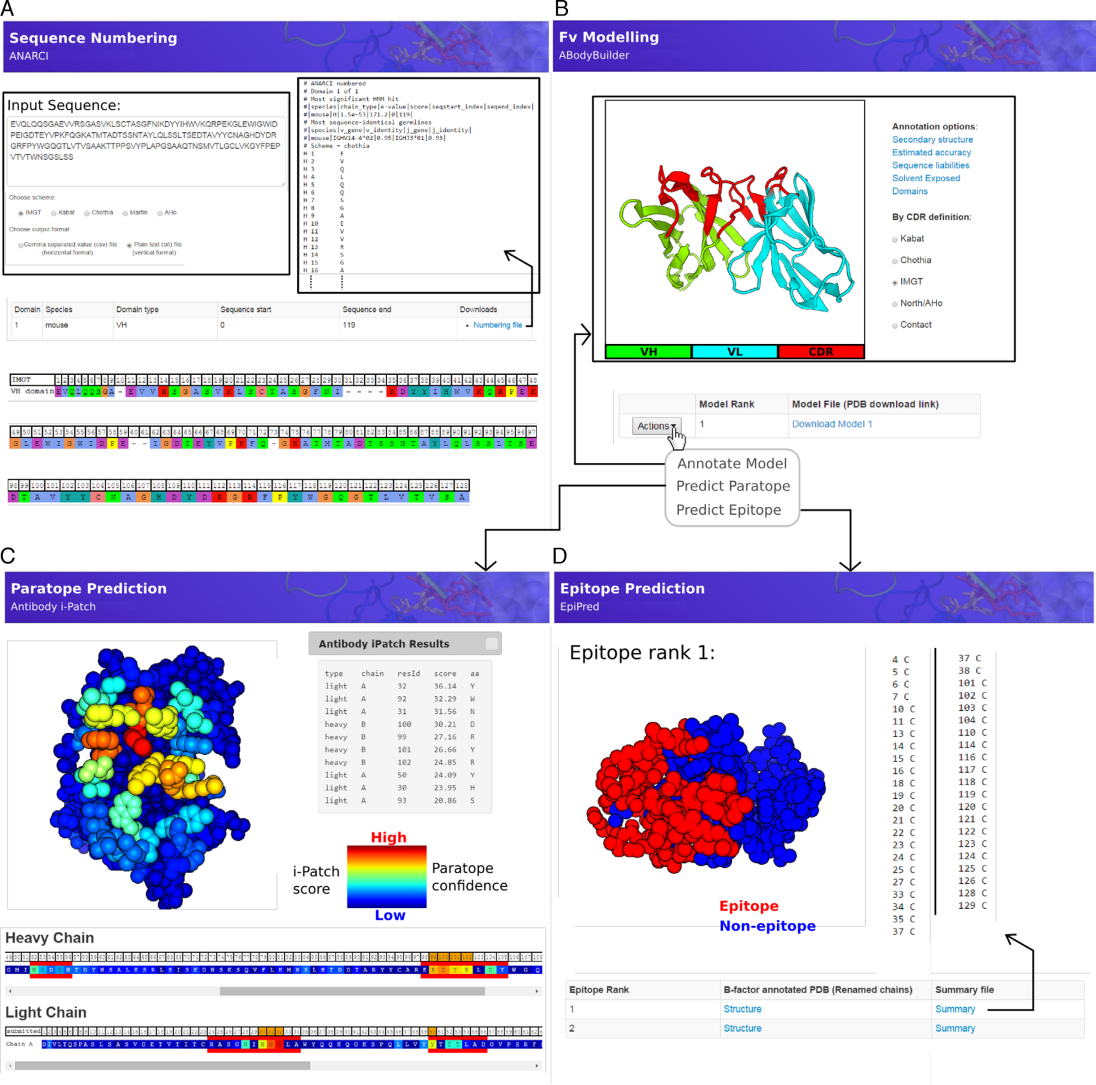
Example outputs of the four main applications provided by SAbPred. (**A**) The ANARCI tool can be used to apply popular antibody numbering schemes to variable domain amino acid sequences. (**B**) The ABodyBuilder tool is an automatic Fv modelling protocol. Once a model has been generated it can be annotated with structural properties such as the location of CDRs residues (shown), the estimated accuracy with which each part of the model has been predicted (Supplemental Figure S2) and those residues that may cause issues for *in vitro* antibody development (Supplemental Figure S3). A model may also be directly used in the (**C**) paratope or (**D**) epitope prediction application. (C) Antibody i-Patch predicts those residues most likely to form the paratope. The antibody structure and sequence are coloured according to the i-Patch score (warmer colours indicate a higher score and confidence that the residue will be part of the paratope). A user may export the top N ranked paratope residues and annotate them with a chosen numbering scheme. (D) EpiPred predicts and ranks patches on the antigen surface that are likely to form the antigen epitope. A list of residues is returned and epitope patches may be visualized on the structure.

The sequence numbering application (Figure 1A) may be used to annotate either a single or multiple antibody variable domain amino-acid sequences. For single sequences a user should paste the raw sequence (e.g. no fasta or clustal header) into the text box. Multiple sequences should be uploaded as a fasta file using the load sequences button.

A user may choose to apply one of five different numbering schemes: Kabat, Chothia, Extended Chothia (Martin), IMGT and AHo. The format of the output file can be chosen as either ‘vertical,’ where the amino acid and the numbering for each residue is reported on a separate line, or ‘horizontal,’ where all submitted sequences are grouped by domain type, aligned according to the numbering scheme and printed as a csv file.

On submission all variable domain sequences are identified. The annotation for each domain is visualized using the JSAV package (31). The numbering files described above are available for download.

### Fv structure modelling and annotation

The antibody Fv modelling application (Figure 1B) accepts as input the heavy and light chain amino acid sequences of the molecule. Sequences should be in raw format (i.e. no header line) and be pasted into the labeled text boxes. A job name and the numbering scheme that will be used to annotate the final model can be specified by the user.

The output page provides the model in PDB format annotated with the numbering scheme of the user's choice, an alignment between the target and templates used in the process and a log of all the parameters used to build the model. A user may use the generated model in the paratope or epitope prediction applications by clicking on the corresponding link in the ‘Action’ menu. Alternatively, further model annotations may be viewed by clicking on the ‘View Model Structure & Annotations’ link.

One may annotate the model with structural properties such as secondary structure, solvent exposure and the CDR regions of molecule according to different definitions. The structural locations of sequence motifs known to cause issues for developability of therapeutic antibody molecules are flagged on the model. A user may toggle each motif on and off and filter by those that are exposed to the solvent. A list of all the identified motifs and their locations may be downloaded as a csv file.

Estimated confidence of model accuracy can also be visualized. The interface allows a user to specify a confidence threshold (e.g. 75% confident) and two thresholds for structural similarity (e.g. within 1Å RMSD and within 2.5Å RMSD) (Supplemental Figure S2). The model will be coloured according to these thresholds and they may be changed dynamically. A user can therefore assess the estimated quality of the model allowing them to gain an intuition as to the extent to which each part of the predicted structure should be trusted for guiding structure-based engineering decisions.

### Paratope prediction

The paratope prediction application (Figure 1C) accepts as input the structure of an antibody and a structure of a protein antigen. If a structure is unavailable for the antibody a model may first be generated using the ABodyBuilder application. The chain identifiers that make up the two molecules must also be provided in the labeled text boxes.

Results are typically returned within a minute and are in the form of a PDB format structure file of the antibody. Here, the B-factor column is replaced with the Antibody i-Patch score. The higher the i-Patch score the higher the likelihood that the residue is in contact with the antigen and is part of the paratope. The structure and sequence of the antibody are coloured by this score and visualized using PV (32) and JSAV (31), respectively. Users may also filter the top N ranked paratope residues and export their details as a list annotated with a chosen numbering scheme.

### Epitope prediction

The epitope prediction application (Figure 1D) takes the same input as the paratope prediction application described above. Again results are typically returned within a minute. The output from EpiPred is a ranked list of the surface patches on the antigen that could form the epitope. For each prediction a list of the residue identifiers that form the epitope is available for download. Each predicted epitope patch may also be visualized using PV.

## CONCLUSION

SAbPred is a web server to make structure-based predictions for antibody engineering and design. It can be used to annotate the sequences of antibodies with different numbering schemes, automatically produce and annotate homology models of antibody Fv regions, predict antibody paratope residues and predict antigen epitope residues. SAbPred is freely available to all users and is available at http://opig.stats.ox.ac.uk/webapps/sabpred.

## Supplementary Material

SUPPLEMENTARY DATA
