# Supplementary material for: SAbPred: a structure-based antibody prediction server
Source: Nucleic Acids Res. 2016 Apr 29;44(Web Server issue):W474–8. doi: 10.1093/nar/gkw361 (PMC4987913; doi:10.1093/nar/gkw361)
Supplement: SUPPLEMENTARY DATA [file supp_44_W1_W474__index.html]

SAbPred: a structure-based antibody prediction server — SAbPred: a structure-based antibody prediction server — SUPPLEMENTARY DATA 

# SAbPred: a structure-based antibody prediction server

## SUPPLEMENTARY DATA

- SUPPLEMENTARY DATA
