# Supplementary material for: SAbPred: a structure-based antibody prediction server
Source: Nucleic Acids Res. 2016 Apr 29;44(Web Server issue):W474–8. doi: 10.1093/nar/gkw361 (PMC4987913; doi:10.1093/nar/gkw361)
Supplement: SUPPLEMENTARY DATA [file supp_gkw361_nar-00269-web-b-2016-File003.docx]

**SUPPLEMENTARY FIGURES**


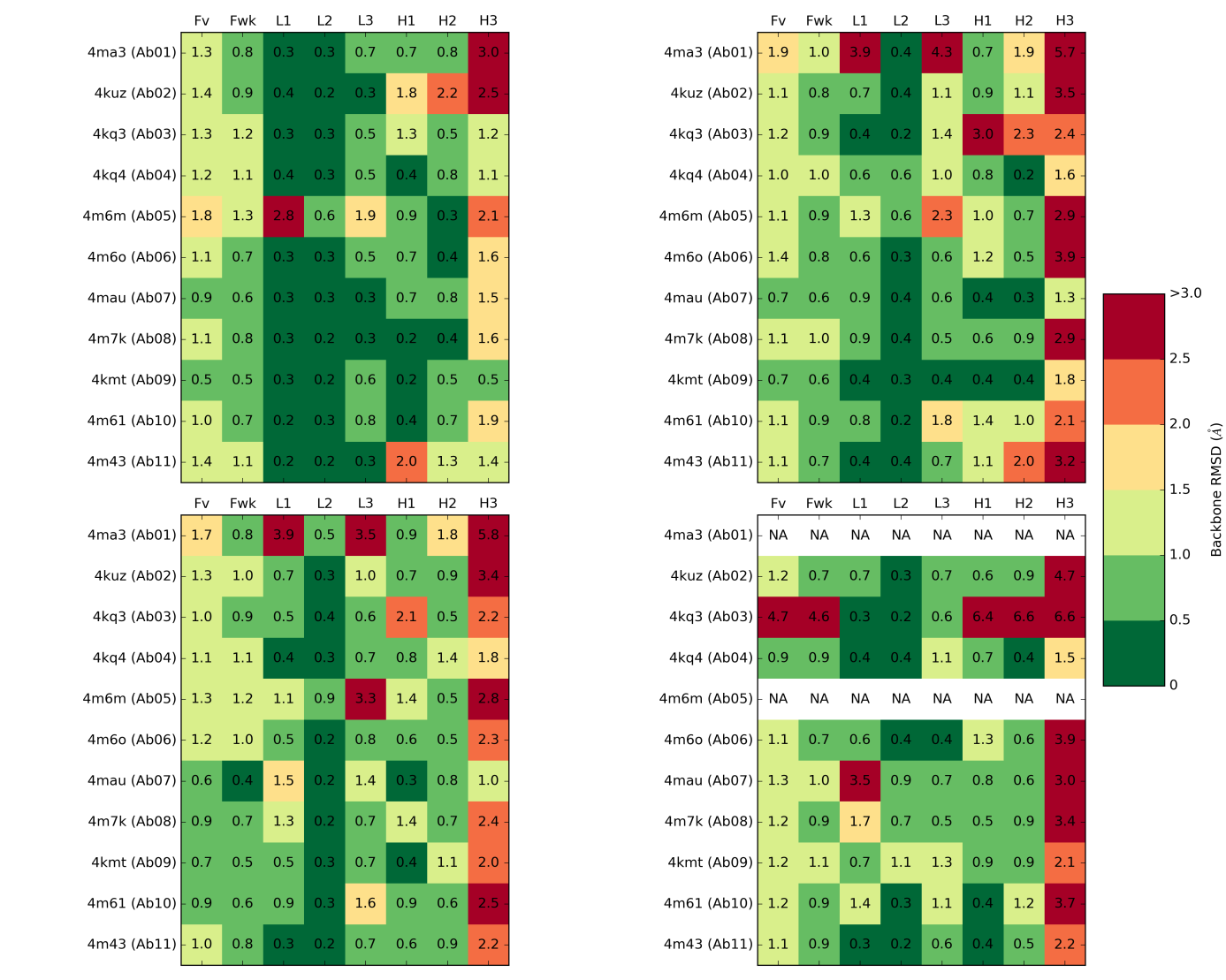


Figure S1. Backbone RMSD heatmap of different methods from the AMA–II competition, including ABodyBuilder (top–left), RosettaAntibody (Weitzner et al., 2014) (top-right), Kotai Antibody Builder (Shirai et al., 2014) (bottom-left), and PIGS (Marcatili et al., 2014) (bottom–right). The RMSD of each region was calculated as described in Almagro et al. (2014). ABodyBuilder was run using only structures that were released to the PDB by 31 March, 2013.


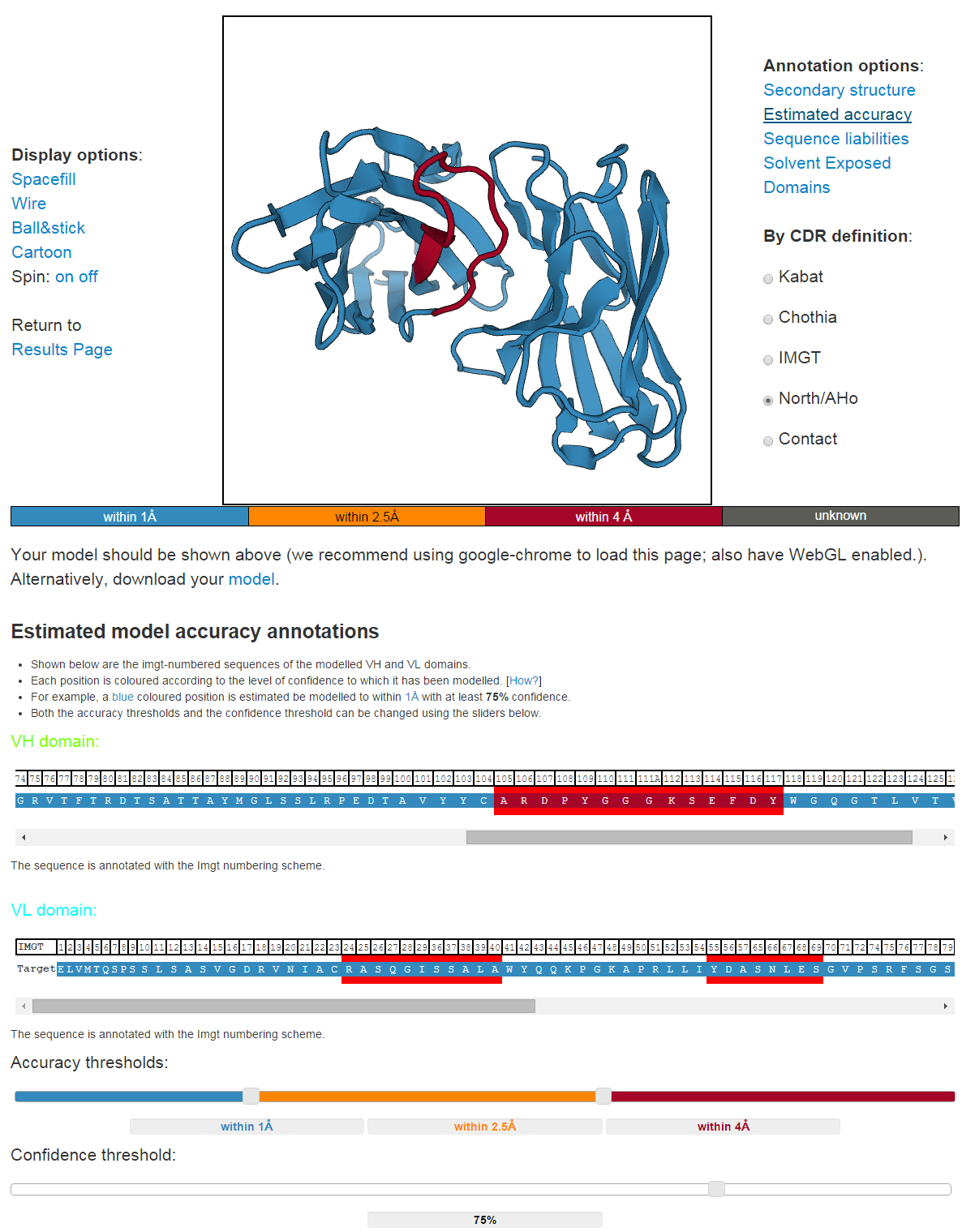


Figure S2: An example output of an ABodyBuilder model annotated with data-driven estimated accuracy. Each part of the modelling process has been extensively benchmarked so that the prediction performance can be estimated based on modelling parameters. For example, the regions in blue have been modelled to within an accuracy of 1Å with a confidence of 75%. For framework regions this means that given the template sequence identity to the target we expect that in at least 75% of cases the coordinates of the backbone and cβ atoms will be predicted to within 1Å RMSD of the crystal structure. For the CDR regions the accuracy estimation is based on the performance of FREAD for the given CDR of that length. A user may change both the confidence and the accuracy thresholds, changing the colouring of both the sequence and the structure.


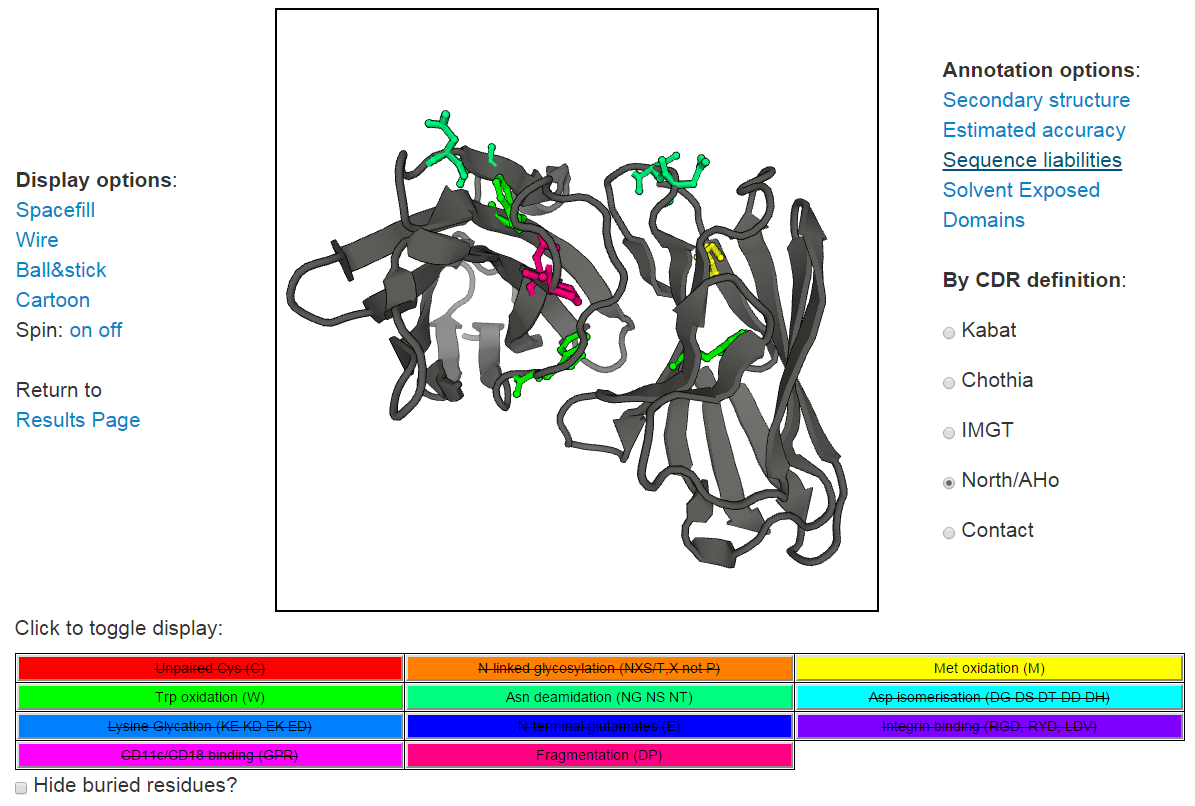


Figure S3: An example output of an ABodyBuilder model annotated with residues that may cause issues for *in vitro* development. Sequence motifs and their associated potential liability have been collected from the literature. When a motif is identified in the sequence it is flagged on the structure for assessment by the expert user. Liabilities may be filtered according to whether they are exposed (>10% relative exposure) or buried. Individual liabilities may also be toggled on or off. A file listing each liability and its location in the sequence is provided for download.
